# Marimo actuated rover systems

**DOI:** 10.1186/s13036-021-00279-0

**Published:** 2022-01-05

**Authors:** Neil Phillips, Thomas C. Draper, Richard Mayne, Darren M. Reynolds, Andrew Adamatzky

**Affiliations:** 1 Unconventional Computing Laboratory, Faculty of the Environment and Technology, University of the West of England, Coldharbour Lane, Bristol, BS16 1QY UK; 2 Centre for Research in Biosciences, Faculty of Health and Applied Sciences, University of the West of England, Coldharbour Lane, Bristol, BS16 1QY UK

**Keywords:** Biomimicry, Bio-energy, Bioengineering, Bio-rover, Soft robotics, Unconventional, Sustainability, Aegagropila linnaei, TRIZ

## Abstract

**Background:**

The potential to directly harness photosynthesis to make actuators, biosensors and bioprocessors has been previously demonstrated in the literature. Herein, this capability has been expanded to more advanced systems — Marimo Actuated Rover Systems (MARS) — which are capable of autonomous, solar powered, movement.

**Results:**

We demonstrate this ability is both a practical and viable alternative to conventional mobile platforms for exploration and dynamic environmental monitoring. Prototypes have been successfully tested to measure their speed of travel and ability to automatically bypass obstacles. Further, MARS is electromagnetically silent, thus avoiding the background noise generated by conventional electro/mechanical platforms which reduces instrument sensitivity. The cost of MARS is significantly lower than platforms based on conventional technology.

**Conclusions:**

An autonomous, low-cost, lightweight, compact size, photosynthetically powered rover is reported. The potential for further system enhancements are identified and under development.

**Supplementary Information:**

The online version contains supplementary material available at (10.1186/s13036-021-00279-0).

## Background

Mounting instrumentation on a mobile platform has many advantages (over stationary) for exploration and dynamic environmental monitoring. Numerous missions in challenging environments have shown conventional rovers have practical issues, such as robustness and autonomous operation in uncertain conditions [1, 2]. Marimo Actuated Rover Systems (MARS) is an unconventional solution which presents a novel, solar-powered, autonomous mobile platform which is a simple but effective solution to some of the detriments of traditional stationary systems.

Nature has evolved processes to harness solar energy and almost all organisms have some method for harvesting it: a common method is via direct conversion of sunlight into energy via photosynthesis. Briefly, this involves the transfer of energy from harvested photons towards a wide variety of life processes via a series of redox reactions within a light-sensitive electron transport system: in green plants, these chemical reactions occur in photosynthetic organelles called chloroplasts, which catalyse the generation of glucose, oxygen and water from input carbon dioxide and water. Although the theoretical maximum photosynthetic efficiency is approximately 36% [3], in practice a harvesting efficiency of around 6% of Photosynthetically Available Radiation (PAR) into biomass is the general best case [4] because the conversion rate drops in full sunlight to avoid damaging the organism [5].

The efficiency of photovoltaic panels varies depending on the type of cells used and operating environment. Panels with crystalline silicon cells typically have a conversion rate of around 15% on Earth [6, 7]. Triple-junction GaAs/Ge cells have a conversion rate of around 23% on Mars [8]. However, the electrical energy usually needs to be stored and converted into other forms (such as mechanical or chemical) which reduces the overall efficiency of conversion.

Combining photosynthesis with human engineering (biomimicry) has enabled novel devices to be prototyped. In our previous study [9] we extended prior research into photosynthesis-powered actuators and biosensors into more advanced systems. In this article we advance the concept of autonomous & self-powered movement, suitable for real applications, and describe a fully-functional prototype rover using a biological organism as its main component.

**Fig. 1:**
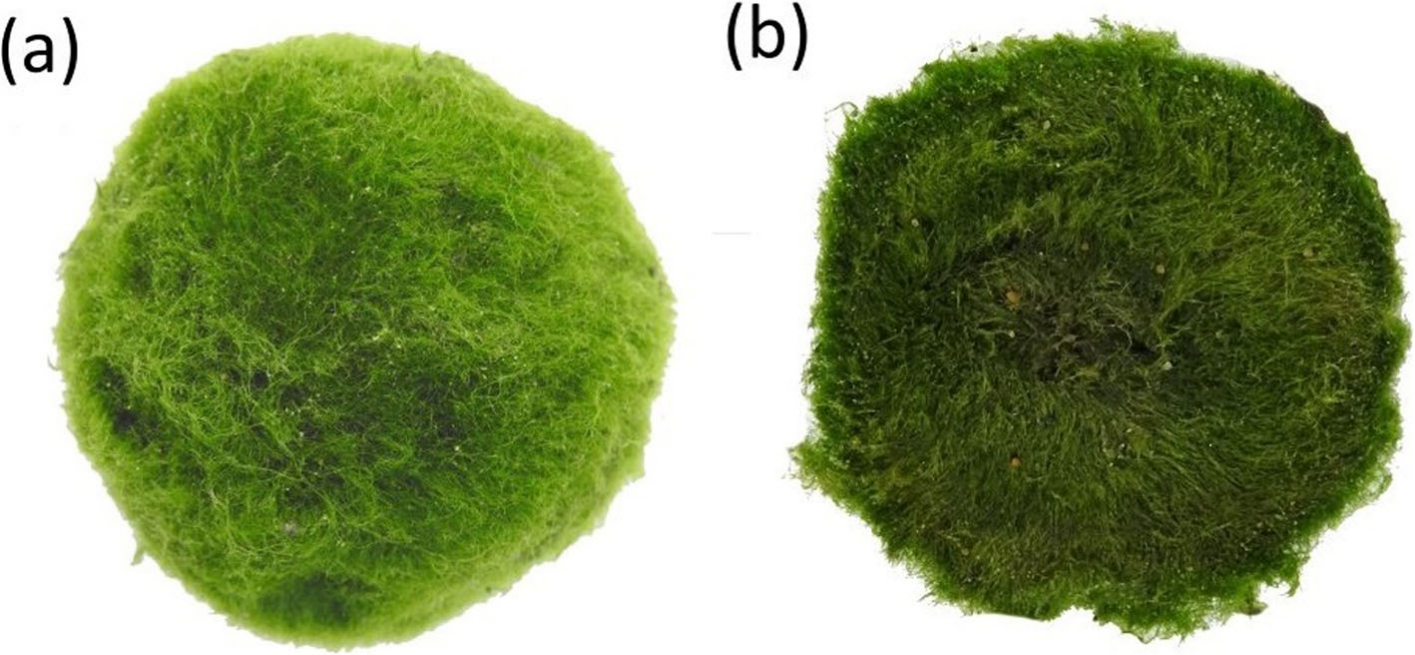
Photographs of Marimo ball **a** intact Marimo **b** cross-sectioned Marimo showing its filamentous nature. This Marimo ball has a diameter of ∼60mm

Experimental testing has demonstrated the algal filaments in *Aegagropila linnaei* balls (commonly called ‘Marimo’) are promising building-blocks for photosynthesis devices. *A. linnaei* balls are spherical objects [10–12], as shown in Fig. 1a, formed by the natural rolling and self-adhesion of filamentous alga in turbulent freshwater currents [13, 14]. Photographs of both an intact Marimo and the cross-section of a Marimo can be seen in Fig. 1.

In the cross-sectional photograph (Fig. 1b), it can be seen that the filamentous nature of the Marimo is continuous throughout. Marimo have been observed to rise and sink in their natural environment due to the generation of gas bubbles which can adhere to their filaments and internal compartments [15].

Converting biomass into electricity or secondary products has been reported by several groups [16]. However, using photosynthesis to *directly* power the movement of rovers (and optionally control) has several advantages. The advancements reported here are steps towards light powered, autonomous, systems which can aid real life missions in challenging environments. For example, environmental monitoring of terrestrial lake bottoms or exploring extraterrestrial environments. We describe here how this technology can be advantageously integrated with conventional electronics, such as sensors/instrumentation and data logging/transmission. As MARS utilises a combination of biological and physical processes it is electromagnetically silent, thus avoiding the additional background noise generated by conventional electro/mechanical mobile platforms which contributes to reducing sensor sensitivity.

The gas generated during the photosynthetic process is used to directly shift the centre of mass of the rover. If the rover is spherical, this facilitates freedom to move in three dimensions. The low density of the gas (0.001 g cm^-3^) compared to water (1.0 g cm^-3^) means the gas rises in the form of bubbles to minimise overall Potential Energy (PE). As the volume of trapped gas increases over time (when illuminated) the peak rotational torque increases until motion is achieved. The volume of the bubble is proportional to the pressure of the water, and therefore the depth of the rover below the surface [17]. Further MARS is able to automatically float/sink (in water) to bypass obstacles.

## Materials and methods

Parts were designed in SolidWorks 2019 (Dassault Systèmes) and 3D printed with PLA on an Ultimaker S5. The print was hand finished to improve surface texture.

The use of Marimo was selected over other options (for example, free floating algae cells) for two primary reasons: (a) Although the body of the Marimo filaments are geometrically constrained their gas bubbles must be able to escape (to achieve motion). Initial testing found the minimum size of gas vent to be an 8mm diameter circular hole or a 6mm width slot for consistent operation (data not shown). Therefore, anything which isn’t ‘held together’ (like intertwined Marimo filaments) can simply escape the enclosure via the vent; (b) If the algae grows quickly it will expand and partly block the vent preventing bubbles from escaping. Therefore, Marimo’s steady growth is well suited to bio-rover for freshwater. For use in sea water, there is a case for replacing the Marimo with slow growing seaweed.

Intertwined algal filaments were evaluated in three forms: spherical (whole Marimo ball), hemispherical (half Marimo ball), and irregular mat (in pentagonal shape).

Artificially-created Marimo balls and mats were sourced from k2aqua UK, kept in large, lidded aquariums in tap water containing 0.1 ml L^-1^ of commercially-available fertiliser containing a source of phosphates and nitrates (TNC Complete, The Nutrient Company, UK). When not in use in experiments, the organisms were exposed to a day/night cycle but were kept out of direct sunlight. The aquarium pump was rated for a recirculating flow rate of 500 L h^-1^. Aquarium water and fertiliser was refreshed every 3 weeks. All Marimo ball & mat cultures and consequent laboratory based experimentation was conducted at room temperature (18^∘^C to 22^∘^C). Experimentation outside was conducted between (5^∘^C to 18^∘^C).

### Geometry of rover units

**Fig. 2:**
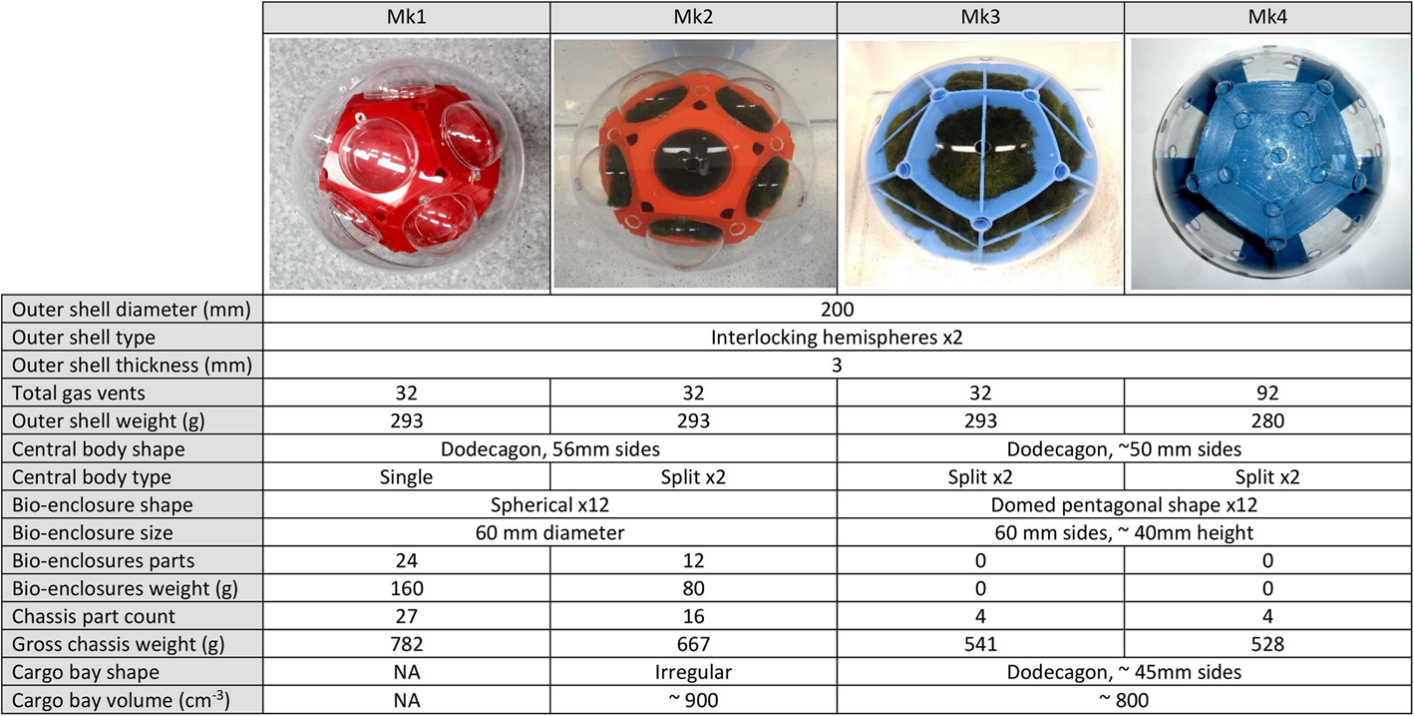
Mk1 with twelve spherical enclosures, Mk2 with twelve half Marimo balls in hemispherical enclosures, Mk3 with twelve irregular Marimo mats formed from partly split balls, in pentagonal shaped enclosures, Mk4 with twelve pentagonal shaped enclosures each with six gas vents

Two geometries of bio-enclosure for algal filaments were selected for prototyping and testing: spherical with Mk1 & Mk2 and domed pentagonal shape with Mk3 & Mk4, see Fig. 2. Derivatives of these shapes were explored to improve performance. Enclosures of each geometric variant were fabricated from a combination of bespoke 3D printed parts and off-the-shelf transparent spheres. Enclosures experimented with included both opaque and transparent sections.

Four strategies were trialled to enhance the rate of bubble generation through photosynthesis. Firstly, by enlarging the diameter of spherical Marimo to increase the illuminated surface area — the diameter was limited inside the enclosures by the Marimo partly blocking the bubble escape vent and restricted water exchange. Secondly, by reducing the diameter of spherical Marimo to allow rotation or movement to expose different filaments to illumination. This was hindered by Marimo floating in the enclosures and blocking bubble escape vents. Thirdly, by changing the illuminated surface from spherical to hemisphere. The diameter of the hemisphere was restricted by packing factor of the chambers inside outer shell [18]. And finally, by changing the geometry of enclosures from spherical (for Marimo ball) to domed pentagonal. The surface area was maximised by using pentagonal geometry to maximise tessellation factor [19].

### Measurement of rate of gas generation

For spherical geometry, two variants were tested: (1a) Marimo ball of ∼60 mm diameter were located inside transparent sphere of 60mm diameter. The size was selected to correspond with previously published measurements [9] (1b) Marimo ball of ∼30 mm diameter in transparent sphere of 60mm diameter. For hemispherical geometry, one variants was tested: (2a) Half (hemisphere) of Marimo ball of ∼60 mm diameter in transparent sphere of 60mm diameter. For domed pentagon Marimo inside regular pentagon (with sides of 60 mm) two variants were tested: (3a) Marimo ball of ∼60 mm diameter partly cut in half and flattened (with two humps) and (3b) pre-made mats (70 mm x 50 mm) trimmed to fit pentagon shape, see S1 in Additional file 1.

The individual enclosures were located inside water-filled, transparent, 2 l glass beakers, under glass funnels (120 mm diameter) attached to syringes (60 ml), such that any released bubbles would rise inside the funnel and be captured. The change in volume of air inside the syringes was recorded while maintaining the same water level inside the funnel. Two funnel rigs were run simultaneously, to ascertain the influence of the geometry on the rate of gas generation. The beakers were filled with tap water containing 0.1 ml l^-1^ of the aforementioned commercially-available fertiliser containing a source of phosphates and nitrates. In the middle of test program, the Marimo were intentionally left without illumination for 32 hour period to act as a control (no gas was collected). For a detailed measurement of illumination level, a PAR sensor was used. Algae filaments were illuminated at 130 Photosynthetic Photon Flux Density (PPFD) by illuminating them with LED growth lamp for 16 hours per day. The PAR light sensor (SQ-120, Campbell Scientific Ltd) was located at the bottom of the glass beaker such that it was illuminated with the test cultures. The sensor’s output voltage was measured with a Fluke 8846A Bench Digital Multimeter.

### Measuring speed of motion

The speed of motion was measured by time lapse photography (using Nikon P900) with two minute time intervals. The rovers were located inside an aquarium (Boyu model EC-600, 66 l capacity, L 600 x W 300 x H 500 mm) filled to level of 400 mm. A black & white checkered pattern (35mm x 35mm) was positioned behind the aquarium. The aquarium’s water pump was left switched off during experiments to prevent recirculating water affecting measurements.

The performance of variants of MARS were investigated with LED grow lamp (Laputa 60LED) set to range of power levels (30%, 40%, 50%, 60% of full power) located at 300 mm wide end of the aquarium. The intensity of the illumination along the rover’s path was measured with a PAR light sensor (SQ-120, Campbell Scientific Ltd). The sensor’s output voltage was measured with a Fluke 8846A. The reduction in illumination with distance from lamp at different power levels is shown in S3 in ESI.

### Measuring autonomous direction

The free motion of Mk2 rover in a transparent plastic container (transparent polypropylene, 145 l, L 710 x W 545 x H 400 mm) was monitored with a camera (Nixon P900). A photograph was taken every two minutes while the rover was illuminated with LED grow lamp on the (545 mm wide) side of the container.

### Measuring obstacle bypassing

The ability of the rover to automatically become positively buoyant, rise, and bypass obstructions was evaluated by positioning a range of barriers across the rovers path: (a) square aluminium bar (H 50, W 50, L 280 mm) and (b) fired-clay building brick (H 104, W 65, L 217 mm). Testing was conducted with LED grow lamp on the (300mm wide) side of the aquarium (Boyu EC-600).

### Measuring rover recovery

A commercially available, combined temperature sensor and data logger (model TempU02, Tzone Digital Technology Ltd) was housed in a waterproof wallet and attached (via ethylene-vinyl acetate thermoplastic) to the middle of a foam sheet (closed cells, ∼4 mm thickness). The foam sheet was cut to: fit inside the cargo bay of Mk4, sized to achieve neutral buoyancy, and allow any trapped gas to escape.

**Fig. 3:**
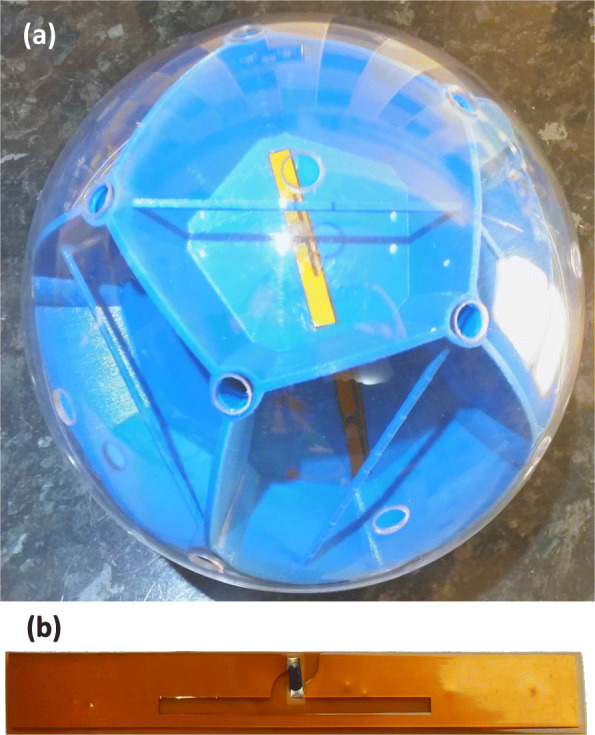
**a** RF reflector mounted on Mk4 rover. **b** Close-up view of model C-19 RF reflector

Six passive RF reflectors (C-19, RECCO Ltd) were uniformly distributed and attached to the central body of Mk4, see Fig. 3a. Each reflector (L 60 x W 10 x H ∼ 0.2 mm and mass 0.16 g) consists of a pair of foil aerials, joined by a diode, see Fig. 3b. The size of the foil pieces are selected to form a resonant circuit such that as one end assumes one polarity the other is of opposite polarity. The diode sees large voltages across it and is operated in its nonlinear region, thus producing harmonics. The whole antenna establishes an oscillating signal along the receiver element [20].

The Mk4 rover was deployed in an outdoor swimming pool (L 4880 W 2440 H 1070 mm). A RECCO transmitter/receiver (model R9, 1.5 W) was used to send out a radio signal at 917 MHz to the reflectors which then double the frequency to 1834 MHz and send it back to the unit [20]. RECCO estimates the maximum range of the detector signal up to 200m in air and 20m through snow depending on conditions [21, 22].

### Measuring electromagnetic emissions

Rover body and Marimo were measured for electromagnetic emissions using Triple Axis, Field Electromagnetic Field (EMF) Meter (model 480826, manufactured by Extech Instruments). The instrument was used on maximum sensitivity (20 *μ*T range) with the probe moved around the test subjects. 10 seconds was allowed for each reading to stabilise.

## Results

### Geometry of rover units

For ease of comparison, all variants were designed with an outside diameter of 200 mm. Applying the principles of engineering design [23] and TRIZ [24] the internal designs of MARS were progressively advanced after each set of measurements evolving Mk1, Mk2, Mk3 & Mk4, see Fig. 2. For example, the chassis part count was reduced from 27 to 4 and the chassis mass from 782 to 528 g. Improving the number and location of gas vents in the shell enhanced performance by reducing trapped gas bubbles, see S4 in ESI.

### Gas generation rate

Groups of algal filaments of different shapes and size were located inside a range of enclosures and illuminated, see S1 in ESI. The daily rates of gas generated by each arrangement are summarised in Table 1.

**Table 1:** Typical gas generation rates for different configurations. S= spherical; P= pentagonal

Enclosure type	Marimo	Rate (cm^3^/d)
(1a) S 60mm dia.	Whole 60mm dia.	4.0
(1b) S 60mm dia.	Whole 30mm dia.	2.8
(2a) S 60mm dia.	Half 30mm dia.	3.5
(3a) P 60mm sides	Two half 60mm dia.	12.4
(3b) P 60mm sides	P Mat 60mm sides	4.3

Sample sizes are, respectively, 3, 16, 13, 32, & 24. See S1 in ESI for details of enclosures

### Speed of motion

**Fig. 4:**
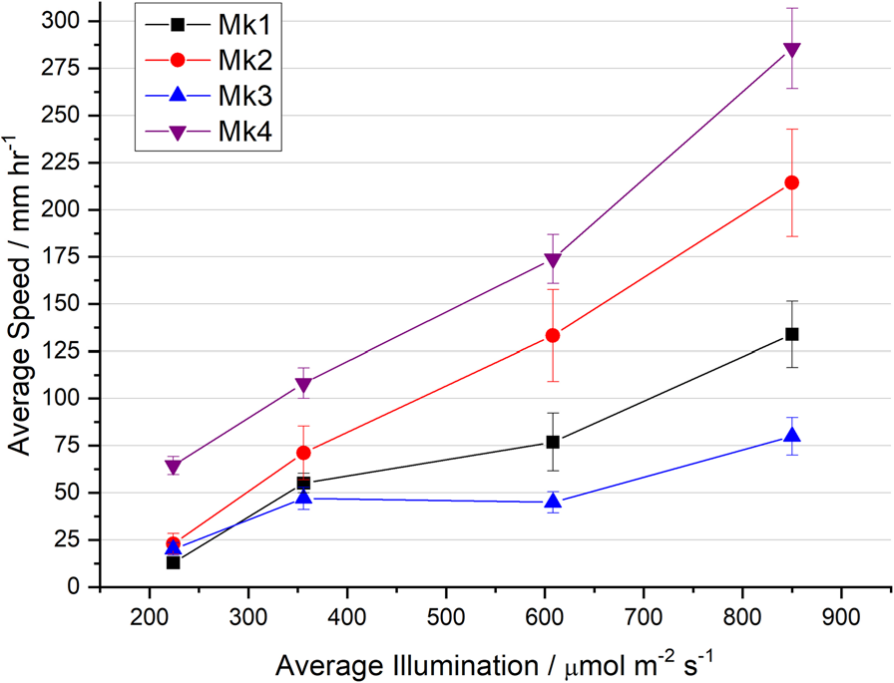
Speed of travel versus illumination level for Mk1, Mk2, Mk3, Mk4 rovers. Error bars indicate standard deviation and measurement uncertainty

Figure 4 shows the speed of travel versus illumination level for Mk1, Mk2, Mk3 & Mk4 rovers.

It was observed that Marimo often have an induction period (several hours) before forming bubbles and movement in a new/refreshed environment. This induction period may be a combination of several factors including: rising concentration of dissolved gases in local water before bubble formation, re-acclimatisation of the organisms to the rapid change in environment, and the state of the Marimos’ photosynthetic systems prior to beginning the experiment [25].

### Autonomous direction

**Fig. 5:**
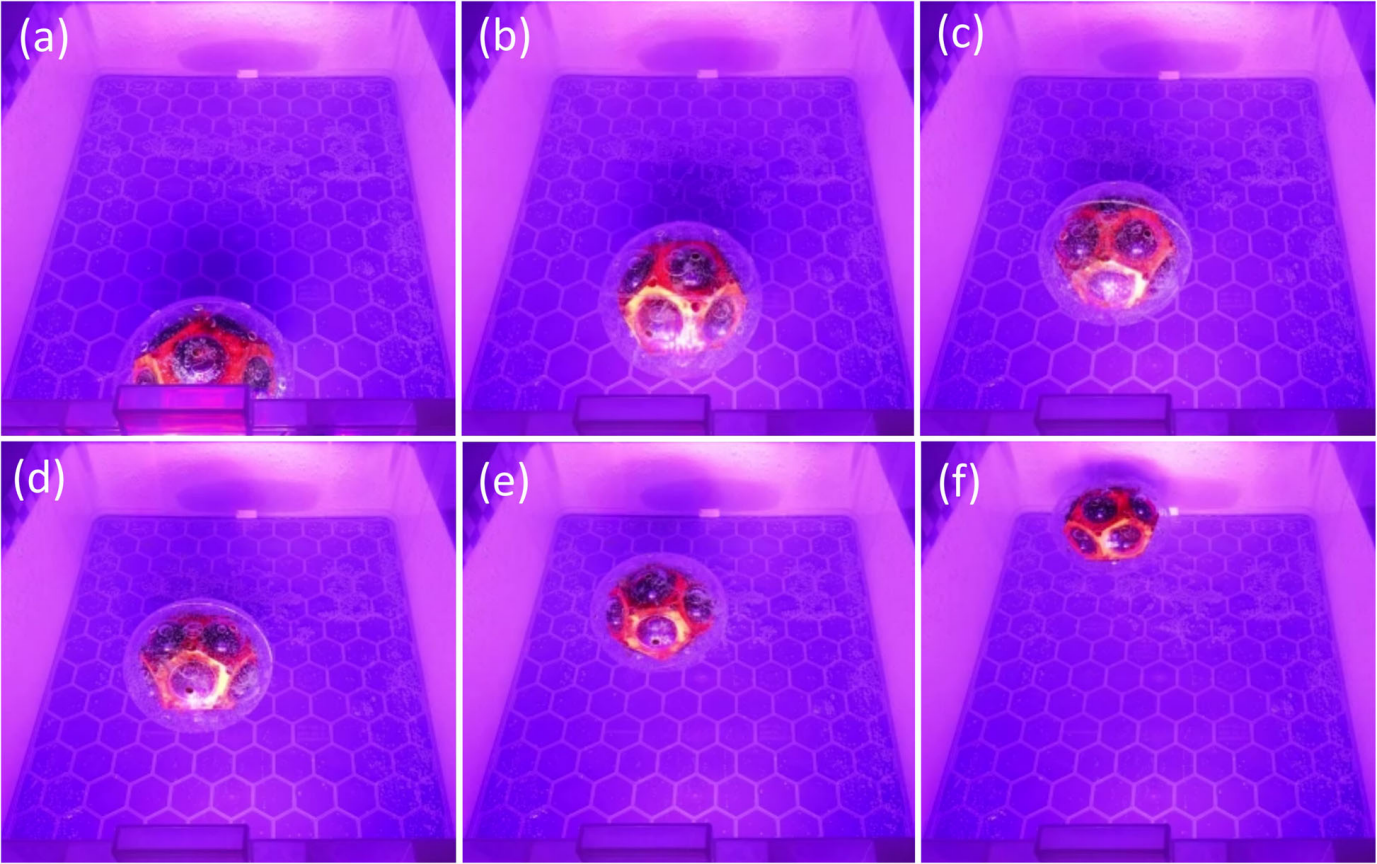
Freeze frame images of Mk2 crossing the container away from LED lamp (Laputa 60LED set to 50% of full power) **a** starting position **b** moving directly ahead **c** moving ahead and to the left **d** moving straight ahead **e** moving straight ahead **f** reaching far end of container

Over ten repeats, the Mk2 rover consistently travelled to the far end of the container away from the light source. The rover often followed a zigzag path with alternating side-by-side motion. It was observed the movement related to the positions of the gas vents. The self guided motion of MARS away from light is shown in Fig. 5.

### Obstacle bypassing

**Fig. 6:**
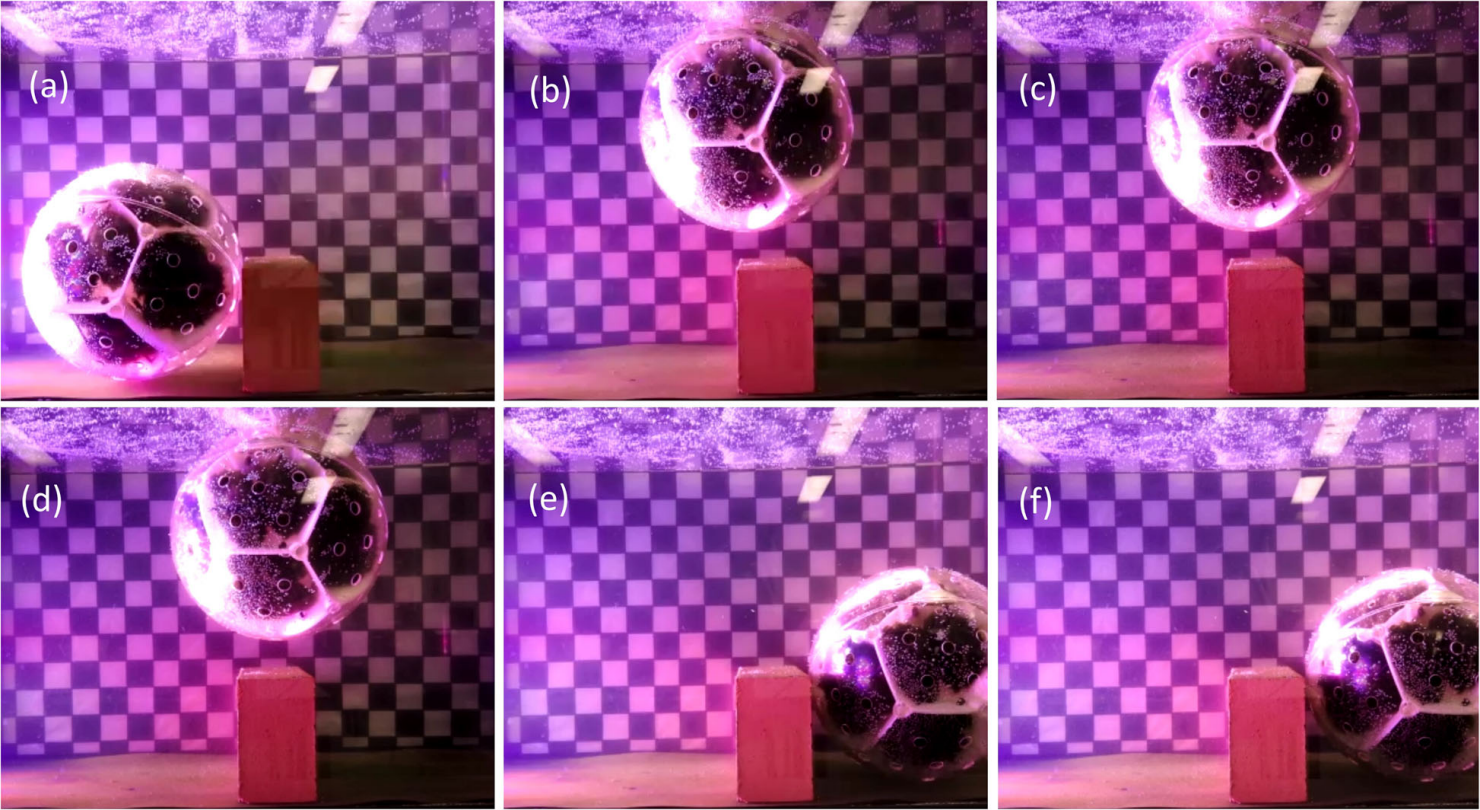
Freeze frame images of Mk4 going over a brick **a** rover unable to continue rolling forward as path is blocked **b** rover automatically becoming positive buoyant **c** rover begins rotate **d** rover passes over obstruction **e** rover sinks **f** rover continues to roll forward

Mk4 consistently travelled across the aquarium bypassing both the aluminium bar (H 50 mm) and brick (H 104 mm) to the far end away from the light source, see Fig. 6.

The rover was observed to operate in three states: (a) physical contact with the bottom; (b) transitioning between the bottom and surface; and (c) floating on the surface.

### Rover recovery

It was not possible to determine the location of the rover on the bottom of the pool (when submerged to a depth of ∼1m) with RECCO handheld transmitter/receiver (R9) and RF reflectors (C-19). However, it could be quickly detected on the surface (or submerged close to the surface, within 50 mm) from a distance of over 20m (the maximum detection range could not be measured due to surrounding buildings blocking line-of-sight).

### Electromagnetic emissions

No electromagnetic emissions were detected from the plastic body of the rovers or Marimo with the EMF meter under a range of illumination levels (dark to bright). Operated on maximum sensitivity (20 *μ*T range) the instrument has an accuracy of ±4*%* Full Scale Deflection +3 digits of 0.01 *μ*T resolution. This indicates MARS is either completely electromagnetically silent or within the instrument’s absolute accuracy of ±0.11 *μ*T in the bandwidth 30 Hz to 300 Hz.

## Discussion

Direct conversion of solar energy into physical motion via biomimicry offers several potential advantages over indirect / multi-stage conversion including: higher reliability, longer operational life, compact size and lightweight. Further, autonomous operation and recovery mode can be integrated.

MARS utilises a wholly biological process (photosynthesis) for energy conversion while bio-inspired robotics are variants of conventional engineering inspired by biological processes. As most biological processes have been optimised through evolution to be as energy-efficient as possible, they are inherently more energy-efficient than conventional engineering inspired by biological processes. While bio-inspired soft robotics aims to produce human-intractable gentle robots, with inspiration from biology. The MARS bio-rover exploits biology, but is not bio-inspired (e.g. not many wheels in nature).

### Geometry of rover units

MARS can be realised in a diverse range of mechanical configurations to suit different operating environments and missions requirements. For example, the hollow core can be utilised to house a variety of instrumentation (e.g. water quality sensors and data logger). In exemplar Mk4, the hollow core is a cargo bay which can accommodate a dodecagon shaped load with 45mm sides and volume of ∼800 cm^3^. The load must adhere to two strict requirements: (a) the core to remain neutrally buoyant, possibly with the addition of positively buoyant material such as foam (in salt water the maximum load can be greater than in fresh water) (b) the centre of mass (of load) must align with the axis of rotation of the rover. For example, Mk4 should accommodate most loads up to ∼0.5 kg while allowing movement for centre of mass to be positioned on axis of rotation.

An array of sensors could be mounted around the periphery, to observe the surrounding environment (on time period or motion basis). Further, limited energy can be extracted from rolling to power instrumentation, see S2 in ESI. Alternatively, power can be provided by a bio-reactor located in the core [26]. Depending on the configuration, photo-voltaic cells can be located between the bio-energy converter modules to provide power to instrumentation and data links. As the surface area of a larger structure increases to the square of its radius, the potential power of the MARS would increase quadratically with its size.

The optimal number/shape of photosynthesis enclosures for a particular environment/mission is a balance between several parameters. In general, the higher the coverage of the rover’s surface with photosynthesis material the greater the amount of solar energy that can be harvested. Lowering the number of divisions was found to reduce efficiency, as bubbles are free to move/rise to the edge of the division without energy harvesting. Testing indicated the number, location and size of the vents has a significant impact on speed and smoothness of rotation. For reliable bubble release the vent needs to be >8 mm in diameter, a ‘lip’ is desirable to retain bubbles until the division reaches the top to maximise energy collection. In most tests, holes of 10 mm diameter were used to vent gas.

For consistent motion it is beneficial for divisions to be of equal (or similar) surface area. For example, the surface of sphere can be completely tilled with equally sized (curved) pentagons forming a regular dodecahedron [19]. Alternatively, the surface can be partly covered with multiple spheres each of equal but smaller diameter (than the main sphere). The former is well suited to hosting two or more half Marimo balls.

### Gas generation rate

It was observed that Marimo balls partly split into two halves, housed in pentagonal shaped enclosures, provided the highest rate of gas generation (∼12.4 cm^3^d^-1^). This is likely due to maximising and optimising both the total available photo-active algae, and the incident angle of said algae to the light. The generated gas, it should be noted, has been previously demonstrated to be photosynthetic in origin [15]. Therefore it is comprised primarily of oxygen, which has the potential to be captured and utilised further.

As a non-planktonic alga, Marimo are not strongly influenced by fluctuations in dissolved oxygen saturation, as indicated by their being adapted for life at a range of depths, i.e. both floating and sessile on lake beds. We did not find published work that specified the tolerance of Marimo to elevated levels of oxygen saturation, but work on unicellular photosynthetic alga (e.g. in the biotechnology field) has shown that they tend to be very permissive to changes in oxygen saturation (more than most other aquatic organisms [27]). In [28], the planktonic alga *Nannochlorposis* only experienced deleterious effects when O_2_ saturation exceeded 75% that of atmospheric air, after which their growth rates reduced due to competitive inhibition of the RuBisCO enzyme, which is involved in photosynthetic CO_2_ fixation.

### Speed of motion

MARS can operate underwater subject to adequate illumination level. The attenuation coefficient of pure water averaged across the photosynthetic spectrum is ∼0.03 m^-1^ [29]. The deepest that light can penetrate water with sufficient intensity for photosynthesis depends on water clarity [30]. For example, plant growth at 70m with just 10% of surface irradiance being available has reported [31]. Being able to operate at depth should enable MARS to operate in lakes with clear water [32]. For example, Antarctica contains 17 clear water lakes with a maximum depth of ∼10m [33]. The photosynthesis material can be selected to operate in either fresh or salt water. The former could be Marimo while the latter could be seaweed.

Depending on the environment, the MARS is capable of operating for prolonged periods of time (months or even years) with minimal resources [13, 34].

The iterations of Marimo based conversion described in the paper can potentially provide a similar order of energy generation (∼22 J) to alkaline AA cell over a year (∼13 J) see ESI. As this system is designed to be long-term and low-maintenance there is clearly an advantage to the bio-rover. Especially when one realises that some missions could be year(s) in length. The iterations described in the paper show incremental improvements in performance. It is highly unlikely that this system has reached its peak at so early a stage in its life, and we anticipate further gains in forthcoming iterations.

### Autonomous direction

Housing photosynthesising material inside multiple, separated, enclosures which are located around the inside surface of a larger structure (e.g. sphere or cylinder) can enable rotation when illuminated. Photosynthesis creates positive buoyancy through gas generation. The net change in buoyancy will actively rotate the structure away from the light source. For ‘continuous’ motion each enclosure requires a gas vent such that the gas can escape when the enclosure moves to the ‘top’ of the larger structure.

If the rover is dynamically balanced such that its centre of mass aligns with the axis of rotation of the rover, the rover’s direction of motion will be primarily determined by direction of illuminations and surface topology. As the direction of illumination is often known (or can be determined by instrumentation) the surface topology can be inferred. The rover’s balance can be adjusted with an additional balance system [35].

The transparency of sections of the outer shell might be varied to control the direction of roll/motion by changing light transmission. For example, electrochromic coating [36] could be added (in sections) to the inside surface of the outer shell. Applying a small potential to particular section(s) in sequence could then reduce the illumination level to particular bio-enclosures. Thus reducing the rate of gas generation which would reduce torque thereby affecting direction/speed of motion. Further, this could also be used to adjust the rover’s gross buoyancy (e.g. darken all sections to create negative buoyancy so the rover sinks when desired).

### Obstacle bypassing

**Fig. 7:**
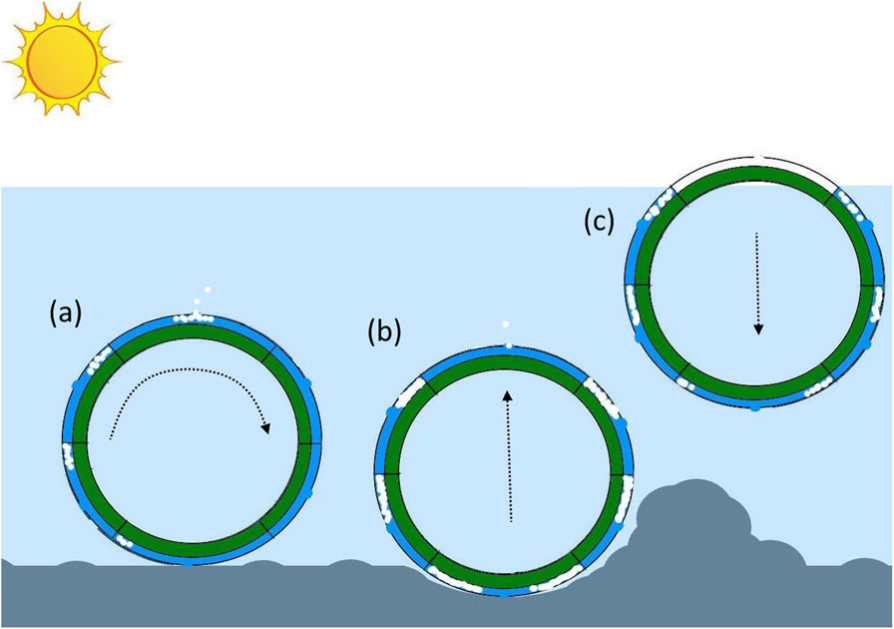
Automatic obstruction bypassing **a** sketch of rover moving forward via rotation **b** sketch of rover unable to rotate as path is blocked by terrain **c** sketch of rover automatically becoming positive buoyant to bypass obstruction

If MARS becomes stuck and unable to roll, gas bubbles are unable to vent from the top. Therefore the pressure of the trapped gas increases, which expels waters from the rover. This reduces the overall density of the rover making it positively buoyant. The rover rises, rotates (moving forward), releases trapped gas, sinks to a new location. Thereby freeing itself to continue on mission, see Fig. 7. This was demonstrated experimentally.

In order to extend the platform’s capabilities, performance can be enhanced by increasing the stiffness and grip of the outer surface of the shell. The ‘effective pitch’ of small bumps can be reduced by increasing the overall external diameter of the rover. The ‘low contact force’ nature of operation is well suited to rough terrain which can damage conventional drive systems.

### Rover recovery

Optionally, the rover can be configured to become positively buoyant for manual retrieval/data collection or data transmission, after a preset time and/or conditions are meet. A prototype of this option is being tested and will be reported in due course. This capability can be combined with RF reflectors for periodic retrieval. Increasing the strength of transmitted signal would allow the rover to be located over greater distance [37]. RECCO have developed a more powerful transmitter/receiver for aerial based searches [22].

The low mass of MARS (Mk4 is under 1kg, see S6 in ESI), compared to equivalent rovers based on electro/magnetic technology, aids recovery and deployment. For example, Mk4 rovers could be automatically deployed by a drone from the air [38] by simply dropping into water and then automatically collected from (the surface) by the same drone (fitted with instrumentation for RF location).

### Adverse emissions

MARS is electromagnetically silent, thus avoiding the background noise generated by conventional electro/mechanical platforms which reduces instrument sensitivity. MARS may therefore be suitable for underwater mine surveying/clearing as the rover is able to transverse where the presence of either electromagnetic emissions and/or ferromagnetic parts is undesirable (e.g. mine detonation). Additionally, MARS’s intrinsic spark-free nature means it is safe to operate in explosive environments.

MARS acoustic emissions underwater (bubbles rising and gentle motion of the body of the rover) are likely to be less intrusive to wildlife than emissions from propellers and electric motors, which have been shown to be highly disruptive to aquatic species [39].

### Applications

The peak power output of Marimo balls is lower than conventional energy cells. However, Marimo balls can potentially continue producing power for much longer compared to limited run time of conventional energy cells, which is an advantage for some applications. The use of passive and ultra-low power sensors can be particularly advantageous with Marimo based rovers.

The total energy and peak power requirements for some exploratory missions can be dramatically reduced in several ways. For example, bio-rovers (including instrumentation) can be configured to be close to neutral buoyancy (slightly negative) so rotational torque for movement is low, combined with moving slowly the required peak power is lower than the peak power of Marimo balls. Moving slowly between two points underwater is inherently more efficient than moving quicker. However, the latter can be necessary with conventional energy cells due to limited run time. Consequently, Marimo balls can enable more efficient overall operation. The nature of our system also makes it less complicated, with fewer points of potential failure. This makes the system more suitable for long-term missions than conventional energy cells. Additionally, the entire system becomes more environmentally friendly when natural power-sources are utilised in place of conventional energy cells.

The high financial cost of conventional (electro/mechanical) rovers has prohibited their use for some applications (e.g. environmental surveying). To lower operating costs of conventional underwater rovers some users even tether their rovers to reduce the risk of losing them. In practice, tethering is only partly successful as ∼10% rovers are lost because of broken tethers [40]. The Bill of Materials for Mk4 rover is provided in S5 of the ESI. The cost of *$*25 is considerably lower than an equivalent mobile platform (based on conventional technology) opening new opportunities for deployment.

If desirable, the rover can be configured into a cylinder shape to provide straight motion or coupled to the cylinder to form a larger structure. The MARS platform is scalable in diameter. This allows it to traverse larger irregularities in surface topology and makes it safer to wild life. Further, the plastic parts can be made bio-degradable to prevent long term harm to the environment, in the unlikely event of the rover being unrecovered.

Potential applications for the MARS platform can be found in situations where speed of operation is not imperative but device longevity is. For example, strategic water sampling and water quality monitoring [41–43], inspection of deep underground mines [44], mediating interactions between underwater animals [45], studies and control of fish groups [46–48], or ecological studies [49]. The principles of operation outlined in this paper can be applied to larger scales which could be used, in turn, to enhance the operation speed.

For non-terrestrial applications, MARS could be transported to location and/or 3D printed on site using solar fused planetary material. To minimise transport volume/mass, and maximise empty rover volume to mass ratio, the rover can be formed from thin/flexible skin which is inflated (like a Zorb ball) to create structural strength [50]. The long life of Marimo (many decades [13, 34]), potential to preserve photosynthetic organisms via cryogenic freezing [51], and option to be grown onsite makes MARS a strong candidate for space exploration. Mathematical models [52] and commuter simulations would be a profitable avenues to explore in the design of space missions. With further development, MARS could complement the current roadmaps for space power technologies for future missions [53].

Several variants of the designs outlined in this paper are currently under consideration:

(1) For underwater applications: the non-magnetic nature of the shell allows for magnetic coupling between the centre and external parts. Some or all of the instrumentation can be located inside a separate positively buoyant chamber above the main sphere. This provides the instrumentation with enhanced access to the surrounding local environment.

(2) For use in murky water: the overall buoyancy can be increased to ensure the rover ‘continuously’ floats on the surface. Adding ‘blades’ to the outside converts rotation into directional motion. Operating close to the surface increases access to irradiance energy for photosynthesis. It also simplifies the tasks of data-transfer and locating the device.

(3) If the vents in the outer shell are modified to release gas while retaining water, water diversion channels and bladder(s) are added, MARS can potentially travel on dry land.

## Conclusions

The principle of using photosynthesis to create autonomous, light-powered rover has been demonstrated. Opportunities for further enhancements to functionality have been identified and are under development. The logistics of using MARS to survey a range of environments is being explored and will be reported in due course. MARS can utilise the continual green energy production of Marimo, and combine it with the system requirements of a particular mission (sensor loadout, buoyancy, etc.).

We have demonstrated that a mobile platform can be built using Marimo. The performance of these example systems has been enhanced by optimisation of the generation/collection of gas bubbles. In design, a balance had to be struck between solar to mechanical energy conversion efficiency versus functionality (in particular, supporting instrumentation and direction control).

## Additional material

Additional file 1 Marimo_Actuated_Rover_Systems (ESI)

## Data Availability

The datasets made and analysed during the current study are available from the corresponding author on reasonable request.
