# Supplementary material for: Marimo actuated rover systems
Source: J Biol Eng. 2022 Jan 5;16:3. doi: 10.1186/s13036-021-00279-0 (PMC8734212; doi:10.1186/s13036-021-00279-0)
Supplement: Supplementary file 1 — Marimo_Actuated_Rover_Systems (ESI) [file 13036_2021_279_MOESM1_ESM.pdf]

Electronic Supplementary Information

Marimo Actuated Rover Systems

Neil Phillips[1,*], Thomas C. Draper[1], Richard Marne[1], Darren M. Reynolds[2], and Andrew Adamatzky[1]

[1]Unconventional Computing Laboratory, Faculty of the Environment and Technology, University of the West of England, Coldharbour Lane, Bristol, BS16 1QY, UK
[2]Centre for Research in Biosciences, Faculty of Health and Applied Sciences, University of the West of England, Coldharbour Lane, Bristol, BS16 1QY, UK
[*]Corresponding author: Neil.Phillips@uwe.ac.uk

S1 Test enclosures

Figure S1 shows the enclosures used for measuring the gas generation rates of individual enclosures.

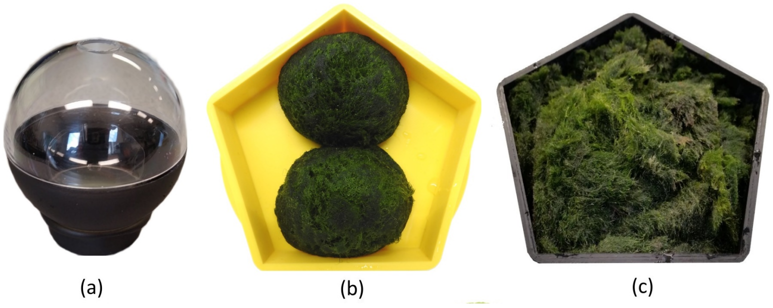

Figure S1: Photographs of enclosures for gas measurements (a) spherical enclosure of 60 mm diameter (b) pentagonal shaped frame with sides of 60 mm containing two half Marimo of $\sim$ 60 mm diameter (c) pentagonal shaped frame with sides of 60 mm containing mat of algal filaments.

S2 Energy calculation

When illuminated, the algal filaments photosynthesis and produce gas bubbles. These bubbles, due to their density being lower than that of water's, rise and become trapped against the inside surface of their individual enclosure. When their enclosure reaches the top of its rotation the bubbles are able to escape. For example, Mk4 with 200 mm diameter and twelve enclosures. During rotation, the bubbles in each enclosure rise by $\sim 170\,\mathrm{mm}$. The typical volume of gas generated by pair of half Marimo is $\sim 12.5\,\mathrm{cm^3\,d^{-1}}$. With a rotational speed of $\sim 0.5\,\mathrm{rev\,h^{-1}}$, half a rotation takes $\sim 1\,\mathrm{h}$ and $\sim 0.5\,\mathrm{cm^3}$ of gas is generated. To approximate the lift, the mass of the (rising) gas bubbles is subtracted from the mass of the water 'sinking' (becoming displaced). There are secondary factors (such as bubble expansion with pressure change/depth).

$$(1.00\,\mathrm{g\,cm^{-3}} \times 0.5\,\mathrm{cm^3}) - (0.001\,\mathrm{g\,cm^{-3}} \times 0.5\,\mathrm{cm^3}) \approxeq 0.5\,\mathrm{g} \tag{1}$$

Potential energy (PE) can be expressed as PE $= mgh$, where m is mass, g is acceleration due to gravity, and h is the distance displaced (or height). Therefore,

$$\mathrm{PE} = mgh = 0.5 \times 10^{-3}\,\mathrm{kg} \times 9.81\,\mathrm{m\,s^{-2}} \times 0.17\,\mathrm{m} \approxeq 0.83\,\mathrm{mJ} \tag{2}$$

The resulting value is based on a single pair half-Marimo completing one revolution (a single Marimo enclosure), therefore the energy can be more properly displayed as $\sim 0.83\,\mathrm{mJ\,rev^{-1}}$ for each enclosure when rotating at $0.5\,\mathrm{rev\,h^{-1}}$. In the case of twelve enclosures, with approximately half of them illuminated at any one time.

$$\text{Total PE} = 0.83\,\mathrm{mJ} \times 6 \approxeq 5\,\mathrm{mJ} \tag{3}$$

Power (P) can be expressed as ΔE the change in energy (Joules) divided by Δt the time taken (seconds):

$$\mathrm{P} = \Delta E \div \Delta t = 5 \times 10^{-3} \div 3600 \approxeq 1.4\,\mu\mathrm{W} \tag{4}$$

Bio rover (with 12 Marimo balls) produces $\sim$ 22 J of energy per year (12 hrs of sunlight light per day). Dry weight of Marimo ball (60 mm diameter) is $\sim$ 8.8 g, therefore weight of 12 Marimo balls is $\sim$ 106 g. For comparison, manganese/alkaline AA cell can output $\sim$ 13 J of electricity energy (battery weight $\sim$ 24 g, plus weight of electric motor(s), gears, etc).

S3 Illumination levels

MARS was tested using a variable power, full spectrum, plant growth lamp, brand name LAPUTA, model number '60LEDS grow light'. Illumination against distance for the light output of the LAPUTA lamp can be seen in Fig. S2.

Photosynthetically Active Radiation (PAR) light sensor (Campbell Scientific Ltd, model SQ-120, measurement repeatability: < 1 %, linear response: $5\,\mu\mathrm{mol\,m^{-2}\,s^{-1}\,mV^{-1}}$) was used to measure illumination levels. The output voltage of the sensor was measured with a Fluke 8846A precision multimeter.

Photosynthetic Photon Flux Density (PPFD) rather than lux was recorded, as the PAR sensor measures how many photons (within the portion of the spectrum useful for photosynthesis) are striking the surface. Every photon is counted the same. By contrast, a lux sensor measures light in the visible spectrum and weights the light best seen by human eyes. Lux sensors tend to measure lower than PAR/PPFD sensors.

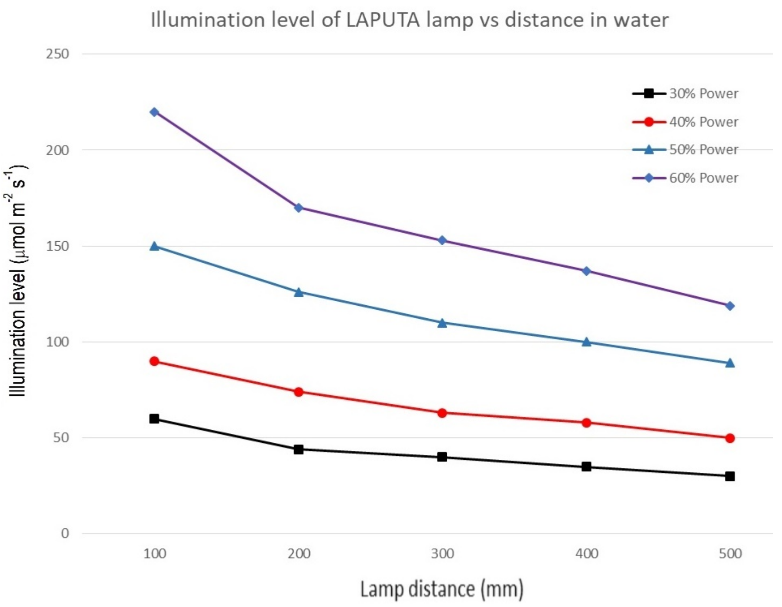

Figure S2: Illumination levels of LAPUTA lamp vs distance in water. Illumination levels attenuated by water

S4 Trapped gas bubble

Fig. S3 shows an example of gas bubble trapped under the outer shell unable to escape when it reached the 'top' of the rover.

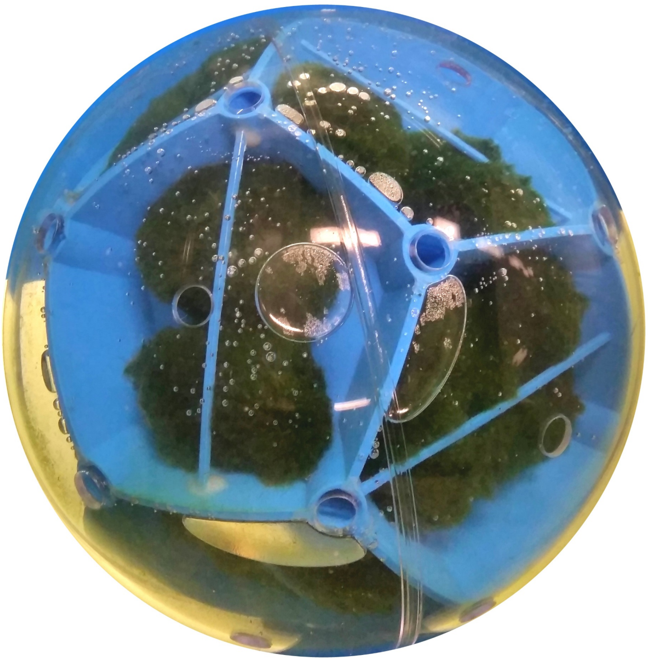

Figure S3: Plan view of Mk3 with gas bubble trapped under shell.

S5 Bill of Materials (BoM)

Table S1 provides a breakdown of the 'one-off' cost for sections of Mk4. Volume pricing would be considerably lower.

Table S1: BoM for Mk4. 3D print files available upon request from corresponding author.

Section	GBP
Hemispherical, interlocking, transparent shell, 200mm diameter, 3mm thickness x2	£6
Plastic body with six pentagonal enclosures (3D printed from ∼115g of PLA) x2	£4
Marimo ∼60mm diameter x12	£15
Total	£25

S6 Rover Mass

Table S2 provides a breakdown of the mass of the Mk4 MARS. Rover mass can be further reduced through optimisation (e.g. thinner wall sections, bespoke rather than off-the-shelf parts).

Table S2: Mass of Mk4. N.B. Marimo ball has a mass of ∼9g if desiccated.

Section	Mass
Hemispherical shell (200mm diameter, 3mm thickness) with vent holes x2	280g
Plastic body (3D printed with PLA) x 2	248g
Marimo ball, ∼60mm diameter, 'wet' x12	460g
Total	990g
